# Tissue culture of two medicinal trees native to Japan

**DOI:** 10.1186/1753-6561-5-S7-P137

**Published:** 2011-09-13

**Authors:** Katsuaki Ishii, Naoki Takata, Manabu Kurita, Toru Taniguchi

**Affiliations:** 1 Forest Bio-research Center, Forestry and Forest Products Research Institute, Japan

## 

Wadatsuminoki (*Nothapodytes amamianus*) is an endangered tree species observed in only Amami Oshima Island located in southern part of Japan. According to the Red list published by Ministry of Environment, it is classified as 1A (Critically endangered) and naturally remaining number is only 20. It contains camptotesin which is used for anti-cancer drugs. Kagikazura (*Uncaria rhynchophylla*) is an medicinal tree species observed widely in Japan and China. It contains alkaloids (rhynchophylline, iso-rhynchophylline, hirstine and so on) which are good for remedy of high blood pressure and dementia. For the purpose of micropropagation and development of basis of useful substance production by cell culture as well as conservation of endangered species, tissue culture procedure was developed for those two species.

Excised shoots of 2 years old seedling of Wadatsuminoki rooted in the 1/2DCR medium containing 3 g/l activated charcoal powder. Newly shoots were induced from in vitro root segments subcultured to 1/2MS medium containing 2uM BAP. This cycle can be used for micropropagation of Wadatsuminoki. We have succeeded in micropropagation by tissue culture of Wadatsuminoki (Figure 1). Callus proliferation from stem or root segments was observed on the 1/2LP medium containing 0.5uM BAP and 1 uM 2,4-D, this subculture cell line may be used for the possible production of secondary metabolites in vitro.

Shoots were induced from stem spine (thorn) of Kagikazura in the 1/2MS medium containing BAP or Zeatin. Regenerated plants were obtained by rooting of these shoots on 1/2MS medium containing 1 uM IBA. Callus induced around the stem segments were continuously subcultured in the fresh 1/2LP medium containing 0.5 uM BAP and 1 uM 2, 4-D. These cell lines can be used for the possible secondary metabolite production and for breeding by somaclonal variation or genetic engineering.

